# The response of mute swans (*Cygnus olor*, Gm. 1789) to vaccination against avian influenza with an inactivated H5N2 vaccine

**DOI:** 10.1186/s13028-016-0255-y

**Published:** 2016-10-22

**Authors:** Beata Dolka, Artur Żbikowski, Izabella Dolka, Piotr Szeleszczuk

**Affiliations:** Department of Pathology and Veterinary Diagnostics, Faculty of Veterinary Medicine, Warsaw University of Life Sciences-SGGW, Nowoursynowska 159c St., 02-776 Warsaw, Poland

**Keywords:** Avian influenza, H5N1, Vaccination, Inactivated vaccine, Mute swans, Racing pigeons

## Abstract

**Background:**

Recent epidemics of highly pathogenic avian influenza (HPAI) produced an unprecedented number of cases in mute swans (*Cygnus olor*) in European countries, which indicates that these birds are very sensitive to the H5N1 virus. The HPAI outbreaks stirred a debate on the controversial stamping-out policy in populations of protected bird species. After preventive vaccination had been approved in the European Union, several countries have introduced vaccination schemes to protect poultry, captive wild birds or exotic birds in zoos against HPAI. The aim of this study was to investigate the immune response of wild mute swans to immunization with an inactivated AI H5N2 vaccine approved for use in poultry. The serological responses of mute swans were assessed by comparison with racing pigeons (*Columba livia*), a species which is characterized by different susceptibility to infection with the H5N1 HPAI virus and plays a questionable role in the ecology of influenza (H5N1) viruses.

**Results:**

Swans were vaccinated once or twice at an interval of 4 weeks. The humoral immune response was evaluated by hemagglutination inhibition (HI) and NP-ELISA. The lymphocyte blast transformation test was used to determine the cell-mediated immune response. Higher values of the geometric mean titer (GMT) and 100 % seroconversion (HI ≥32) were noted in double vaccinated swans (1448.2) than in single vaccinated swans (128.0) or in double vaccinated pigeons (215.3). Significant differences in HI titers were observed between swans and pigeons, but no variations in ELISA scores were noted after the booster dose. Immunization of swans had no effect on the proliferative activity of lymphocytes.

**Conclusions:**

The inactivated H5N2 vaccine was safe and immunogenic for mute swans and pigeons. Vaccination may have practical implications for swans kept in zoos, wildlife parks or rehabilitation centers. However, challenge studies are needed to prove the efficacy of the H5N2 AI vaccine.

## Background

Mute swans (*Cygnus olor,* Gm. 1789) of the order *Anseriformes* and other wild birds of the orders *Anseriformes* and *Charadriiformes* are important natural reservoirs of low pathogenic avian influenza (LPAI) viruses. LPAI viruses of subtypes H5 and H7 can evolve into HPAI (highly pathogenic avian influenza) viruses once introduced into poultry. In contrast to LPAIV, HPAI viruses are rarely detected in wild birds [1–4]. The first known report discussing the possible role of swans in the epidemiology of avian influenza was dedicated to whistling swans in Japan [5]. Following a massive HPAI outbreak in wild geese in 2005, the Qinghai-like HPAI virus H5N1 (clade 2.2) continued to spread across three continents, resulting in devastating losses in the poultry industry. Mute swans were the most frequently affected species of wild birds, particularly in Europe [3, 6–8]. Subsequent investigations revealed the sensitivity of swans to infections and variations in the pathobiology of the HPAI H5N1 virus [3, 7, 9]. Several studies have demonstrated that mute swans could be vectors of HPAI H5N1 over short distances, and that they represent a new avian host in HPAI virus ecology [6, 7]. Mute swans were recognized as a source of infection in the first global cases of HPAI H5N1 infections passed by birds to humans [10]. Further studies demonstrated that the opportunities for swan-to-human transmission of H5N1 were extremely rare [11]. In AI surveillance programs, mute swans were an effective indicator species (sentinel birds) in the epidemiology of HPAI [3, 7]. Since 2010, HPAI H5N1 viruses have become enzootic and evolved dynamically to form multiple H5 HA clades [4, 12]. Between 2014 and 2015, various HPAIV H5 subtypes of several clades have spread rapidly and globally in poultry and wild birds [12, 13]. Furthermore, mute swans (Sweden 2015) and other swan species were affected by the novel reassortant H5N8 virus [8].

Unlike swans, pigeons were long regarded as a species that is highly resistant or minimally susceptible to infection with HPAIV [14–16]. However, according to the recent literature, pigeons’ susceptibility to H5N1 virus and their role in AI epidemiology have been questioned [16, 17]. Avian influenza is still an unsolved problem for the racing pigeon industry, mainly due to a lack of detailed regulations. The restrictions put on poultry in connection to suspected and confirmed HPAI outbreaks also apply to domestic pigeons. Thus vaccination may be a solution to avian influenza problem in racing pigeon industry.

The global strategy for the control and eradication of HPAI involves strict administrative procedures, including confinement, stamping-out and pre-emptive culling [18, 19]. The application of radical solutions to healthy, valuable, endangered and protected species of birds (held in captivity) was met with high opposition for ethical, social, and environmental reasons [20]. Pursuant to the provisions of Council Directive 2005/94/EC, AI vaccination was approved in EU as an additional measure disease control and an alternative to pre-emptive culling. The vaccination strategy should be carried out only in combination with biosecurity measures [21–23]. In the EU, commercial inactivated AI vaccines were licensed for use only in chickens and Pekin ducks, but they were also shown to be effective in other bird species [24–26]. It should be noted that only individual species of wild birds kept in captivity can be considered for AI vaccination. Many studies demonstrated that vaccine-induced antibody responses in birds were determined by the species, vaccine type or dose [25, 27]. However, little is known about vaccination of swans and pigeons against HPAI H5N1 [26, 27, 42, 48].

In view of the above, the aim of this study was to evaluate the immune response of mute swans immunized against AI with an inactivated vaccine. The serological response of swans was compared with that of racing (domestic) pigeons (*Columba livia f. domestica*) immunized with the same vaccine. Pigeons were compared with mute swans based on differences in the susceptibility of the two species to H5N1 HPAIV.

## Methods

### Mute swans

Eleven free-living mute swans were captured in early spring in central Poland. Immature swans of both sexes (2-year-old males n = 4, 36.4 %; females n = 7, 63.6 %) were marked with ornithological rings and transported to the Agricultural Experiment Station of the Warsaw University of Life Sciences. They were placed in individual boxes and exposed to the natural light–dark cycle. Feed and water were provided ad libitum.

### Vaccine

Birds were immunized with the Nobilis® Influenza H5N2 vaccine based on an inactivated, whole avian influenza virus (A/duck/Potsdam/1402/86, European strain) inducing an HI titer of ≥6.0 log_2_ (MSD Animal Health, Boxmeer, Netherlands). The vaccine strain has 90 % nucleotide sequence homology to the HA gene of the H5N1 HPAI field strain A/turkey/Turkey/1/05; clade 2.2 (1530 base pairs, including basic cleavage site), and 92.4 % homology based on the amino acids sequence [30]. The vaccine was chosen based on its specific subtype, availability, licensing considerations, safety and efficacy data for poultry and other birds, and DIVA (Differentiating Infected from Vaccinated Animals) guidelines [18, 28–30]. The Nobilis® Influenza H5N2 vaccine is commercially available and has been approved by the European Commission and the European Medicines Agency [31].

## Experimental design

### Vaccination of mute swans

Mute swans were divided into two experimental groups (D1, D2, n = 8) and one control group (C, n = 3). Each group contained birds of both sexes: D1 (n = 4): 1 male and 3 females; D2 (n = 4): 2 males and 2 females; C (n = 3): 1 male and 2 females. Birds were manually restrained for vaccine administration. Group D1 swans were vaccinated once (day 0). Swans from group D2 were vaccinated twice: on day 0 and 4 weeks later (4-week interval). The dose was adjusted for body weight. Birds were given a dose of 1 ml. The vaccine was administered subcutaneously to the nape of the neck. The control group was not vaccinated.

### Vaccination of racing pigeons for comparative analysis

Four young racing pigeons aged 3–12 months were vaccinated twice according to the schedule for D2 mute swans. The vaccine was administered subcutaneously in the dorsal region of the neck. Each pigeon (body weight >400 g) received a dose of 0.5 ml. Pigeons were kept at the Small Animal Clinic of the Faculty of Veterinary Medicine at the Warsaw University of Life Sciences (SGGW). They were placed in individual cages (450 mm × 900 mm) with ad libitum access to water and feed, temperature of 22–25 °C and humidity of 50–70 %. The birds were exposed to 10 h of light per day.

### Safety monitoring

During the study, swans and pigeons were observed twice daily and were weighed before the first vaccine administration and then every 7 days. After immunization, vaccine tolerance and any side effects, in particular localized reactions at the injection site, were recorded. Birds were also observed for adverse effects [25].

### Serological tests

Blood samples from the medial metatarsal vein were collected 5 times: before the first vaccination (0) and in weeks 2, 4, 6, and 8. At these dates, the presence of H5 subtype-specific antibodies in serum samples was determined by the hemagglutination inhibition test (HI). The HI test was performed according to standard procedures using 4 HAU of commercially available H5 heterologous antigen (A/chicken/Belgium150/99soncke99/150v6, H5N2) (GD Animal Health, Deventer, the Netherlands). HI titers were expressed in log_2_, where 4 log_2_ (HI ≥16) was the minimum positive titer [15, 23]. The geometric mean titer (GMT), seroconversion (SR) and protection rates (PR) were determined with the use of methods described in previous studies [25, 27, 32]. According to Bertelsen et al. [32] the SR and PR were expressed as the percentage of birds with titers higher than or equal to 16 and 32, respectively. Similarly to other authors, we assumed for the purpose of this study a HI titer ≥32 could be considered protective [25, 32].

The presence of AI type-specific antibodies was determined by NP (nucleoprotein)–blocking ELISA (FlockChek AI MultiS-Screen Ab Test Kit, IDEXX Laboratories, Inc., Westbrook, Maine USA). ELISA results were analyzed using xChek® ver. 3.3 software (IDEXX Laboratories, Inc., Westbrook, Maine USA). The mean S/N ratio (sample to negative control ratio) and coefficient of variation (% CV, standard deviation divided by the mean) were presented. The S/N cut-off was set at <0.5 to identify antibody-positive samples.

Before vaccination (day 0), serum samples were examined for the presence of NDV antibodies in the HI test to reduce the risk of possible cross-reactions and false positive results. The NDV antigen, LaSota strain (GD Animal Health, Deventer, the Netherlands) was used in HI [23, 29].

### Evaluation of cell-mediated immunity by the Lymphocyte Blast Transformation Test

Lymphocytes from heparinized peripheral blood sampled from swans were separated by the gradient centrifugation method using Gradisol L (Aqua-Med, Poland) and were washed in supplemented Eagle’s medium (Biomed Lublin, Poland). Cells were adjusted to 2.0 × 10^6^ cells/ml in Eagle’s medium with fetal calf serum, and 100 µl from each well was transferred to 96-well plates. Lymphocyte cultures were stimulated by two non-specific T cell mitogens: phytohemagglutinin (PHA) and concanavalin A (ConA) (Sigma-Aldrich, USA), as well as a specific stimulator: inactivated AIV H5 antigen (A/Chicken/Belgium150/99, LPAI H5N2). Each mitogen (100 µl) was added in triplicate at a final concentration of 5.0, 2.5 and 1.25 µg/ml for PHA and ConA and with HA activity of 1/8 and 1/16 for Ag H5. The cultures were incubated for 72 h at 41 °C. Radiolabeled ^3^H-thymidine (40 MBq/ml) (Lacomed, Czech Republic) was added for the final incubation of 24 h at 41 °C. Controls included unstimulated labeled and unlabeled cells. The general procedure was adopted based on the literature [33–35]. After incubation, the cells were deposited on glass fiber discs with a cell harvester (Skatron, Norway), and the amount of incorporated radioactivity was measured with the Wallac 1414 WinSpectral liquid scintillation counter (PerkinElmer Life Sciences, Wallac Oy, Turku, Finland). T-cell proliferative activity was expressed by the stimulation index (SI). Proliferation was regarded as significant at SI ≥2 [36].

### Virus isolation

Before vaccination, cloacal swabs from swans and pigeons were collected in tubes containing a liquid medium: sterile phosphate buffered saline—PBS (BIOMED, Lublin, Poland) with an antibiotics (Antibiotic Antimycotic Solution 100× , Sigma, USA). Clarified supernatant fluids from swabs were inoculated (0.2 ml) in embryonated 11-day-old chicken eggs. The eggs were incubated at 37 °C for 72 h and candled daily. Amnio-allantoic fluid was harvested and tested in the hemagglutination assay (HA). HA-negative samples (derived from swans) were passaged three times using undiluted amnio-allantoic fluids as inoculum. One passage was performed for samples derived from pigeons. Three eggs per sample were used in every passage [23, 29].

### Molecular tests

RNA was isolated from amnio-allantoic fluids and cloacal swabs using the QIAamp Viral RNA Mini Kit (Qiagen, Germany). The presence of AIV was tested by matrix (M) gene-based Real-Time RT-PCR using the OneStep RT-PCR Kit (Qiagen, Germany) [37]. For more specific detection of the H5 subtype (including Eurasian isolates), the H5 gene was amplified by Real-Time RT-PCR and RT-PCR [38, 39]. The presence of NDV was checked by RT-PCR [40]. Two inactivated H5N1 antigens (A/whooper swan/Germany/R65-2/06, A/turkey/England/250/07) and the NDV antigen were used as positive controls.

### Statistical analysis

The results were processed in Statistica 10.0 software. Group means were compared by the t-test for variables with normal distribution and in the Mann–Whitney U test for other distributions. Three or more groups of variables were compared against normal distribution by one-way analysis of variance for completely randomized or repeated measures and Fisher’s multiple comparison procedure. The Kruskal–Wallis test was used to compare non-normal distributions. The results were expressed as arithmetic means and standard deviation (SD). Geometric means were calculated for selected variables. The results were regarded as significant at P < 0.05 and highly significant at P < 0.01.

## Results

### Safety

Mute swans and pigeons showed no clinical signs of disease before vaccination. All swans were in good health, and their average body weight was determined at 8.3 ± 1.2 kg (8.8 ± 0.5 kg in males, 8.0 ± 1.4 kg in females).

No adverse effects were observed. The final body weight of the swans decreased by 1.1 kg during the study period, but the differences in the average body weight of experimental and control group birds were not statistically significant (P > 0.05). The behavior of vaccinated swans did not differ from that of control swans. Cases of mortality or injuries due to handling, catching and manual restraint were not observed.

### Serological tests

Before vaccination (day 0), swans and pigeons tested seronegative for AIV/H5 in HI and NP-ELISA. The unvaccinated control remained seronegative throughout the entire 8-week experiment. Serum samples tested before the experiment, gave negative results for NDV in HI.

### Post-vaccination HI titers in mute swans

**Fig. 1:**
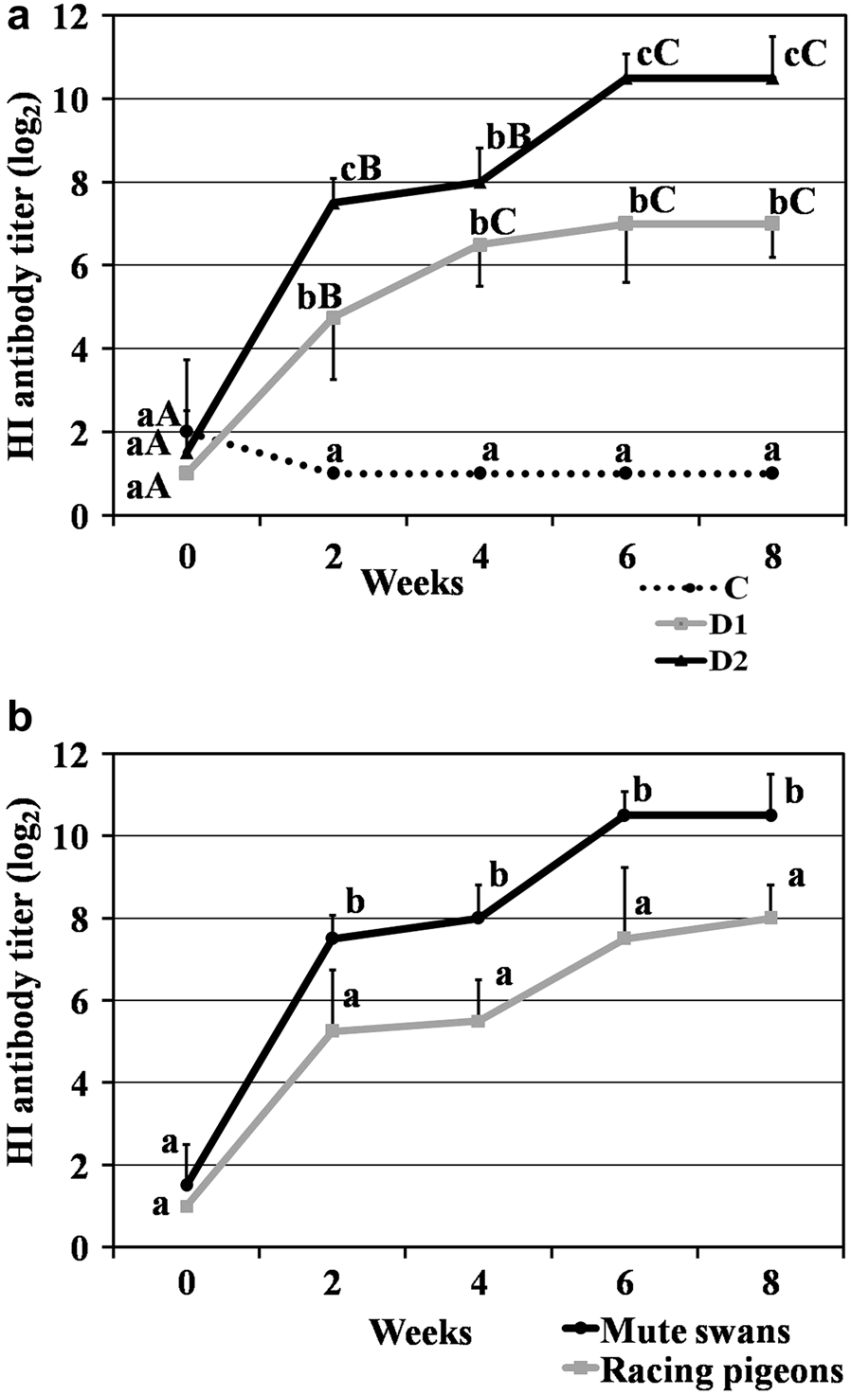
**a** Mean log_2_ HI titers against the H5N2 antigen in control (C) and vaccinated mute swans (D1, D2). **b** Mean log_2_ HI titers against the H5N2 antigen in mute swans (group D2) and racing pigeons. Interpretation: HI ≥ 4 log_2_ (16) positive, HI < 4 log_2_ (16) negative. *Small letters* (*a*, *b*, *c*) denote significant differences in log_2_ HI titers between groups in successive weeks based on the results of the Kruskal–Wallis test. *Capital letters* (*A*, *B*, *C*) denote significant differences in log_2_ HI titers between weeks (*0*, *2*, *4*, *6*, *8*) within groups based the results of the on Kruskal–Wallis test

Vaccine antibody responses in the HI test are shown in Fig. 1a. Statistically non-significant (NS) differences were found between groups in week 0. In weeks 2, 4, 6 and 8, the mean log_2_ HI titers in D1 and D2 birds were significantly higher (P < 0.01) than in the control group. Beginning from week 2, highly significant differences (P < 0.01) in HI titer values were observed in groups D1 and D2. However, 25 % of vaccinated swans were negative in the HI test two weeks after the administration of the first dose. Four weeks after the first vaccination the overall GMT was 152, and SR (HI ≥ 16), PR (HI ≥ 32) reached for the first time 100 %. After the administration of the booster dose, antibody titers in group D2 increased significantly by 2.5 log_2_ and were higher (P < 0.01) than in group D1. The highest antibody titers were noted in weeks 6 and 8 in groups D1 (7.0 log_2_) and D2 (10.5 log_2_). At the end of study (week 8), GMT was approximately 11-fold higher (GMT 1448.2) in swans administered a booster vaccine than in swans that were vaccinated only once (GMT 128.0). In week 8, SR and PR reached 100 % (HI ≥ 16 and ≥ 32) in D1 and D2 swans. No statistically significant differences were found in group C during the study.

### Post-vaccination HI titers in racing pigeons

The results of the HI assays are shown in Fig. 1b. The titers were positive between week 2 (after the first vaccine dose) and the end of the experiment. In week 4, the overall GMT was determined at 45.3, and SR reached for the first time 100 %. The earliest sampling point when PR reached 100 % was two weeks after booster dose (week 6). The highest antibody titer in pigeons (8.0 log_2_), 100 % SR and PR were noted in week 8. Significant differences (P < 0.05) in log_2_ HI titer values between pigeons and swans were noted in weeks 2 and 6, whereas highly significant differences (P < 0.01) were observed in weeks 4 and 8. In general, antibody titers in D2 swans were higher (P < 0.05) than in pigeons after the first and second vaccination. No significant differences were found between groups at week 0.

### ELISA results in mute swans

**Fig. 2:**
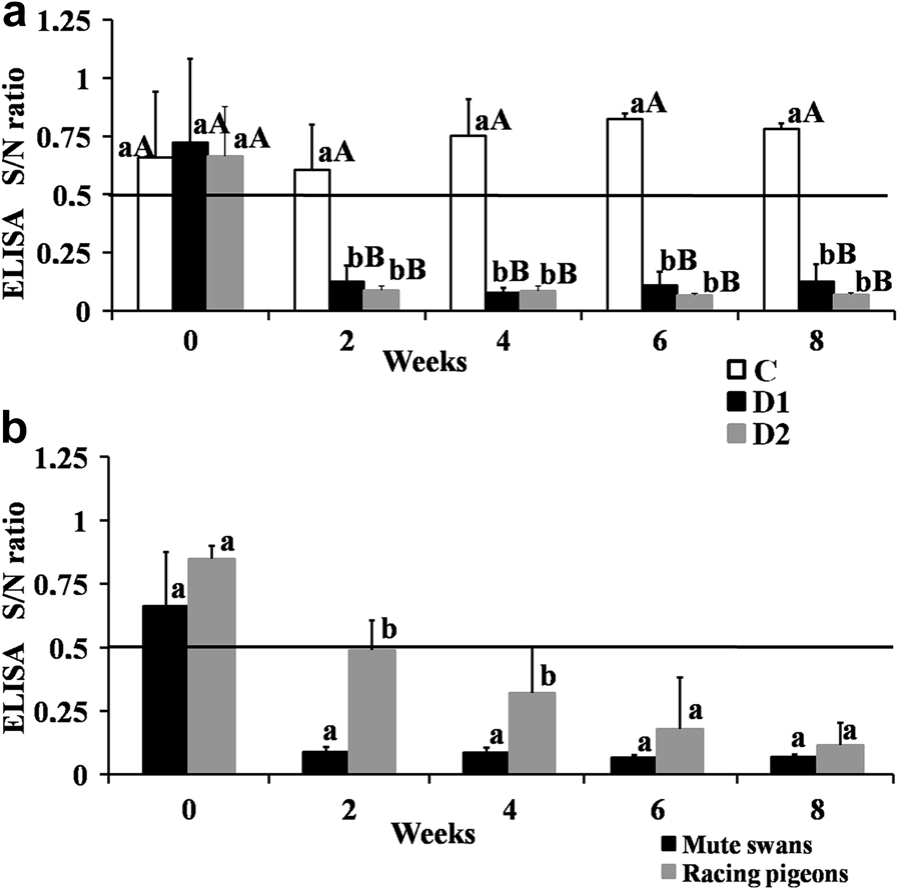
**a** Mean values of the S/N ratio in ELISA for control and vaccinated mute swans (groups C, D1, D2). **b** Mean values of the S/N ratio in ELISA for mute swans (group D2) and racing pigeons. Interpretation: cut-off: S/N = 0.5, S/N < 0.5 positive, S/N ≥ 0.5 negative. *Small letters* (*a*, *b*) denote significant differences in the values of the S/N ratio between groups in successive weeks based on the results of Fisher’s multiple comparison procedure. *Capital letters* (*A*, *B*) denote significant differences in the values of the S/N ratio between weeks (*0*, *2*, *4*, *6*, *8*) within groups

The results of the NP-ELISA are shown in Fig. 2a. No differences were found between the groups in week 0. After vaccination, highly significant differences (P < 0.01) were noted in S/N values in groups D1, D2 relative to the values noted before vaccination and the control group. In weeks 2, 4, 6 and 8, highly significant differences (P < 0.01) in the S/N ratio were noted between C, D1 and D2, whereas no significant differences were observed between D1 and D2. The mean S/N values after the first vaccination were determined at 0.106, CV 48.8 % (week 2) and 0.082, CV 24.2 % (week 4). The mean S/N values in group D1 reached 0.110 (CV 45.5 %) in week 6 and were somewhat higher in week 8 (S/N 0.126, CV 52.4 %). The booster dose induced a minor decrease in S/N to 0.066 (CV 13.4 %) in group D2 in week 6. In group D2, S/N values were determined at 0.070 (CV 10.9 %) in week 8. Statistically non-significant (NS) differences were found in group C during the study.

### ELISA results in racing pigeons

Vaccinated pigeons tested positive for NP antibodies in ELISA (Fig. 2b). After the first vaccination, S/N values were determined at 0.488 (CV 20.8) in week 2, 0.321 (CV 48.6 %) in week 4, and they were significantly higher than in swans (P < 0.01 in week 2 and P < 0.05 in week 4). After the administration of the booster vaccine, the lowest S/N value (0.115, CV 53.9 %) was observed in week 8. A tendency towards higher S/N values was not observed in pigeons (in weeks 2, 4, 6 and 8). Statistically non-significant differences in S/N values were found between swans and pigeons (in weeks 6 and 8) after the second vaccine dose.

### Lymphocyte blast transformation test

The results of the lymphocyte blast transformation test showed no difference between control and vaccinated animals (Table 1).

**Table 1:** In vitro proliferative responses (SI ± SD) of peripheral blood lymphocytes after stimulation with PHA, ConA mitogens and H5 antigens in mute swans from control and experimental groups (C, D1, D2)

Mitogen concentrations [µg/ml] or antigen (HA titer)	Week^NS^	SI ± SD values in groups of mute swans^NS^		
		C	D1	D2
PHA 5.0 µg/ml	0	1.29 ± 0.13	0.68 ± 0.78	1.31 ± 1.10
	4	0.90 ± 0.06	1.26 ± 0.18	0.96 ± 0.13
	8	1.22 ± 0.06	1.20 ± 0.36	1.20 ± 0.09
PHA 2.5 µg/ml	0	1.14 ± 0.13	0.77 ± 1.02	1.43 ± 0.33
	4	1.16 ± 0.23	0.55 ± 0.14	0.90 ± 0.62
	8	1.41 ± 0.09	1.73 ± 0.38	1.53 ± 0.16
PHA 1.25 µg/ml	0	1.59 ± 0.44	1.5 ± 0.58	1.88 ± 0.28
	4	1.26 ± 0.36	1.01 ± 0.18	1.21 ± 0.74
	8	1.17 ± 0.86	2.41 ± 1.49	1.09 ± 0.34
ConA 5.0 µg/ml	0	1.27 ± 0.18	1.46 ± 0.06	1.74 ± 0.59
	4	0.92 ± 0.07	1.98 ± 1.02	1.21 ± 0.34
	8	1.14 ± 0.11	1.26 ± 0.45	1.84 ± 0.51
ConA 2.5 µg/ml	0	1.44 ± 0.33	1.85 ± 0.46	1.67 ± 0.51
	4	1.02 ± 0.16	2.18 ± 1.08	1.58 ± 0.91
	8	1.70 ± 0.37	1.86 ± 0.63	3.16 ± 1.46
ConA 1.25 µg/ml	0	1.82 ± 0.26	1.41 ± 0.14	1.15 ± 0.48
	4	1.06 ± 0.18	0.99 ± 0.64	1.29 ± 0.67
	8	1.93 ± 0.87	1.69 ± 0.00	3.17 ± 1.51
H5 (HA 1/8)	0	0.96 ± 0.05	1.29 ± 0.09	0.80 ± 0.58
	4	1.01 ± 0.07	0.56 ± 0.25	0.90 ± 0.00
	8	1.24 ± 0.22	1.18 ± 0.42	1.2 ± 0.15
H5 (HA 1/16)	0	1.04 ± 0.38	0.83 ± 0.43	1.03 ± 0.46
	4	0.77 ± 0.10	0.45 ± 0.20	0.79 ± 0.00
	8	1.27 ± 0.26	1.60 ± 0.81	1.31 ± 0.11

*NS* no statistically significant differences based on analysis of variance and Fisher’s multiple comparisons in SI between weeks and groups for a given mitogen

### Virus isolation

Amnio-allantoic fluids from eggs inoculated with the medium from cloacal samples collected before vaccination, produced negative results in the HA test.

### Molecular tests

All samples (cloacal swabs and amnio-allantoic fluids from eggs inoculated with the medium from cloacal samples) tested negative for AIV in Real-Time RT-PCR (M gene-based) and in the H5 subtype-specific PCR assays. NDV RNA was not detected by RT-PCR.

## Discussion

Several schedules of AI vaccination for exotic birds have been proposed [41–44], but specific antibody titers with confirmed protective levels for mute swans and racing pigeons have not yet been identified. The present study was carried out to analyze the immune responses of wild mute swans vaccinated with an inactivated AI H5N2 vaccine licensed for use in chickens in the EU. To the best of our knowledge, this is the first study to investigate the serological responses induced by AI vaccination in mute swans. The antibody response of swans was compared with that of racing pigeons.

According to the literature, inactivated H5N2 vaccines are well tolerated by domestic waterfowl and zoo birds [25, 41]. The vaccine used in the present study was safe for swans and pigeons. Our findings corroborate the results of studies which demonstrated that AI vaccines approved for use in poultry induced an immune response in non-target avian species [27, 30, 41]. Other authors noted 100 % SR in *Anseriformes* or in black swans after the first dose of an inactivated H5N2 vaccine [41, 44]. In this study, seroconversion was not observed in all swans after the first vaccination, which could point to variations in early response. Our results are in agreement with the findings of other authors who demonstrated differences in immune responses within the same bird species [32, 41, 45]. In our study, the overall GMT in mute swans after the first dose was higher than the known GMT values for black swans and other *Anseriformes* [26, 41, 44, 45], and it was lower than reported in *Accipitriformes, Phoenicopteriformes* [27, 44]. Based on the literature, the differences induced by vaccination are observed between and within taxonomic orders. It could be assumed that single dose of the vaccine may induce relatively high antibody titers in mute swans. However, the type of H5 vaccine strain may have impact on the antibody responses [25, 27, 30].

Similarly to other studies, the effect of a booster dose was clearly demonstrated in this study [22, 25, 42, 44]. High seroconversion (100 %) was induced by booster dose of an inactivated H5N2 vaccine in black swans and other *Anseriformes* [41, 44], whereas lower seroconversion was also reported [27]. We observed dissimilar kinetics of the anti-NP and anti-HA antibodies after vaccination. The differences in NP-ELISA scores between D1 and D2 were not statistically significant throughout the study (week 2, 4, 6, 8). The findings indicate, that booster dose of vaccine did not have a significant impact on the increase of anti-NP titers, in contrast to anti-HA titers. The results highlight the role of anti-HA antibodies in the immune response to AI vaccination.

Elevated GMT in mute swans supported the observations made in black-necked swans, coscoroba swans and trumpeter swans, but differed from the values reported in whistling swans where GMT was low [30]. The H5N2 vaccine used in this study and H5N9 vaccines induced lower overall GMT and seroconversion in different species of *Anseriformes* than in mute swans [26, 27, 30, 32]. However, the differences in HI titers can be attributed to species-specific factors, vaccination schedules or differences in HI assays.

A clear relationship between HI titers and protection against AIV of the same HA subtype [2, 22] was reported in chickens, but it was not noted in mute swans. Studies of ducks demonstrated that vaccines characterized by high protein homology of HA1 with the challenge strain (89–100 % of amino acid similarities) delivered the best protection results [46]. This study relied on the assumption that vaccination in mute swans would produce similar results. The strains of HPAI viruses subtype H5N1 circulating at that time in Poland belonged to clade 2.2 [15]. Based on the high percentage (90–92.4 %) of homology of the vaccine strain with strains H5N1 (clade 2.2), we suggested that both antigenic and genetic distance between vaccine strain and circulating field strains in Poland can be considered the same (or very similar). Antigenically drifted avian influenza viruses decreased the effectiveness of commercial poultry vaccines, but not all antigenic variants of HPAIV H5N1 evaded vaccine-induced immunity in birds [46, 47]. Some studies revealed that genetically more distant vaccines could protect ducks against infection with H5N1 viruses [46]. In our study, a potent antibody response was observed in mute swans, but its role in conferring immunity against heterologous viruses needs to be further investigated.

In this study, booster vaccination was needed for all racing pigeons to produce titers of ≥32. The overall GMT was similar to that of rock pigeons vaccinated with the H5N6 vaccine [48], but was lower than obtained by H7N1 vaccine [42]. Unlike in this experiment, the same H5N2 vaccine induced lower antibody titers and seroconversion in *Columbiformes* [27, 30]. In this study, species-specific differences in vaccine-induced antibody titers were noted between mute swans and racing pigeons. The overall GMT in pigeons was approximately sevenfold lower than in swans. Our findings are consistent with the results reported in *Anseriformes* which developed stronger immune responses to inactivated vaccines than *Columbiformes* [26, 30, 48]. Selected species-specific factors, such as the role played by a given species in avian influenza epidemiology, AIV ecology and its susceptibility to HPAIV infection, could influence on the vaccine-induced antibody titers.

In our study, the vaccine promoted the development of both subtype H5 and type A influenza specific antibodies (anti-NP antibodies) in swans and pigeons. The significance of the antibody response to NP-induced by vaccination has not been fully elucidated. Anti-NP antibodies are not neutralizing, but according to recent reports, they could play a role in the promotion of cellular immunity [49].

Immunization of mute swans had no effect on the proliferative activity of lymphocytes. Our results were in agreement with data demonstrating that immunization of ducks with an inactivated vaccine did not increase the cellular response [34, 36]. Higgins et al. [34] suggested that ducks could have detectable levels of serum antibodies when the cell-mediated immune response is not detected in the lymphocyte transformation test. Further research into cellular immune responses in *Anseriformes* is needed to validate this hypothesis.

## Conclusions

The present study demonstrated that immunization of mute swans with an inactivated H5N2 vaccine is safe and induced a strong humoral immune response. Humoral responses in swans were higher than in pigeons, but the induced antibody titers exceeded the levels presumed to confer immunity in poultry [2, 25, 27, 32]. A booster dose is recommended to enhance and maintain antibody titers. Our results could have practical implications for vaccination strategies for captive swans in zoos, wildlife parks or rehabilitation centers. Under extraordinary circumstances, vaccination of racing pigeons may be a useful strategy to save these birds from indoor confinement and pre-emptive culling (especially valuable individuals) due to the potential risk of AI outbreak. It could allow to continue the long-term breeding work and thus avoid economic losses. The challenge studies are needed to prove the efficacy of the H5N2 AI vaccine, in particular against the new H5 virus variants, and to formulate conclusive recommendations for immunization.

## Abbreviations

AI: avian influenza
AIV: avian influenza virus
APMV-1 (NDV): avian paramyxovirus serotype 1 (Newcastle Disease Virus)
GMT: geometric mean titer
HAU: hemagglutinating units
HI: hemagglutination inhibition
HPAI: highly pathogenic avian influenza
SI: stimulation index
LBTT: lymphocyte blast transformation test
PR: protection rate
SR: seroconversion rate
